# Clinical outcome of FLAG-IDA chemotherapy sequential with Flu–Bu3 conditioning regimen in patients with refractory AML: a parallel study from Shanghai Institute of Hematology and Institut Paoli-Calmettes

**DOI:** 10.1038/s41409-018-0283-5

**Published:** 2018-08-06

**Authors:** Ling Wang, Raynier Devillier, Ming Wan, Justine Decroocq, Liang Tian, Sabine Fürst, Li-Ning Wang, Norbert Vey, Xing Fan, Didier Blaise, Jiong Hu

**Affiliations:** 1 Shanghai Institute of Hematology, Department of Hematology, Blood and Marrow Transplantation Center, Collaborative Innovation Center of Hematology, Rui Jin Hospital, Shanghai Jiao Tong University School of Medicine, 18F/OPD Bldg 197 Rui Jin Road II, 200025 Shanghai, China; 2 Department of Hematology, Program of transplantation and cell therapy, Program of leukemia, Centre de recherche en Cancérologie de Marseille (CRCM), Institut Paoli-Calmettes, Aix Marseille University, 232 Boulevard Sainte Marguerite, 13273, Marseille, CEDEX 9 France; 3 Shanghai Clinical Research Center (SCRC), (Feng Lin International Centre), 18F Bldg A, 380 Feng Lin Road, 200032 Shanghai, China

**Keywords:** Acute myeloid leukaemia, Stem-cell therapies

## Abstract

The purpose of the study was to evaluate the feasibility of conditioning regimen with sequential chemotherapy (FLAG-IDA), followed by Fludarabine (5 days) + Busulfan (3 days) by parallel analysis of patients with refractory acute myeloid leukemia (AML) from two transplantation centers in China and France. A total of 47 refractory AML with median bone marrow blast of 35% (1–90%) and median age at 42 years (16–62) were enrolled. Thirteen patients received peripheral stem cell transplantation (HSCT) from HLA-matched sibling donor, while 18 and 16 from unrelated or haplo-identical donors, respectively. With a median follow-up of 24.3 months (1–70), 13 patients relapsed at a median time of 5.1 months (2.2–18.0) and 24 patients died due to relapse (*n* = 12) or non-relapsed mortality (NRM, *n* = 12). The estimated 3-year RR and NRM were 33.5 ± 5.7% and 25.7 ± 4.2%, respectively. The estimated 3-year overall survival (OS) and event-free survival (EFS) were 43.8 ± 7.8% and 42.3 ± 7.8%. In multivariate analysis, age (<40) and low bone marrow blast were associated with better EFS, while no difference was observed between the two centers. The patients enrolled in study were unselected, representing typical patients' population of refractory AML, and primary data demonstrated the feasibility of sequential conditioning regimen.

## Introduction

The prognosis of patients with refractory acute myeloid leukemia (AML) is poor. Primary treatment failure or primary refractory AML is usually defined by failure to achieve 50% reduction in blast numbers or achieve a complete hematologic remission (CR) after two courses of induction chemotherapy, while refractory-relapsed patients were defined as failure to obtain CR after relapse [1–3]. For these patients, the overall response to salvage the chemotherapy of high-dose cytosine arabinoside (Ara-C)-based regimen, including combination of anthracycline, fludarabine, or gemtuzumab–ozogamicin, remained <30–50%, and most patients eventually relapsed within 3–6 months, even though, clinical remission (CR) can be achieved [4, 5]. Allogeneic stem cell transplantation (allo-HSCT) is considered as the only curative therapy for patients with refractory AML. Based on the conventional conditioning, high relapse rate (RR) and/or non-relapse mortality (NRM) lead to dismal overall survival (OS) as low as 10–20% [6, 7].

To tackle the problem, sequential transplant approach was developed, which combines intensive salvage chemotherapy to decrease the leukemia cell burden with reduced intensive conditioning regimen (RIC). The first report of sequential FLAMSA strategy (fludarabine, intermediate dose Ara-C, and amsacrine followed by 4 Gy total body irradiation, cyclophosphamide, and anti-thymocyte globulin (ATG)) achieved an improved OS and leukemia-free survival (LFS), particularly for patients with only 1–2 cycles of chemotherapy before transplantation. However, for patients who received three or more cycles of chemotherapy, the overall outcome remained unsatisfactory in terms of high RR and NRM [8]. We developed a new transplantation protocol with a salvage chemotherapy composed of granulocyte colony-stimulating factor (G-CSF), fludarabine, Ara-C, and idarubicin (FLAG-IDA) given before transplantation. With a 7-day interval, reduced-intensity conditioning with fludarabine and 3-day busulfan (Flu–Bu3) were given usually at the hematological nadir of previous FLAG-IDA chemotherapy. In our previous phase II clinical study, we tested the feasibility and outcome of this regimen in patients with refractory AML and demonstrated a significantly reduced relapse and improved OS compared with allo-HSCT with conventional conditioning regimen [9].

Based on this intense transplantation protocol, the feasibility of the strategy remains to be determined. We are particularly interested to compare the real practice of refractory AML treatment in two centers in terms of patients’ characteristics (median age and GVHD prophylaxis strategy) and to confirm the feasibility of this transplantation strategy. Thus the study was designed to compare the overall outcome between the two centers and then to confirm the feasibility of this sequential transplantation approach in refractory AMLs. These parallel studies were developed at Rui Jin hospital (RJH, Shanghai, China) and Institut Paoli-Calmettes (IPC, Marseille, France) for patients with refractory AML to evaluate the overall transplantation outcome and confirm the feasibility of the sequential regimen, combining FLAG-IDA, before allo-HSCT, with Flu–Bu3 regimen in a heavily treated, but relatively young patients population from RJH and also in a more elderly, although less heavily treated patients’ series (IPC) with PT-CY as GVHD prophylaxis.

## Patients and methods

### Study design and inclusion criteria

This retrospective study included a total of 47 patients diagnosed with refractory AML who underwent allo-HSCT between 2011 and 2016. The study was approved by the two participating center’s institutional review board. Informed consent from patients and donors was obtained. Patients aged between 16 and 60 years were eligible for the study if they had a refractory AML defined by either: (i) primary induction failure (PIF) with less than 50% reduction in bone marrow blast, with >15% residual blasts after one cycle of induction chemotherapy, or persistence of >5% leukemic blasts in the bone marrow after two induction chemotherapy, or persisting hypoplasia defined by a hypocellular bone marrow and incomplete reconstitution of cell counts in the peripheral blood (absolute neutrophil count < 0.5 × 10^9^/L or platelet count < 50 × 10^9^/L) at day 100 after starting chemotherapy; (ii) early relapse, with the duration of first remission <6 months; (iii) relapse disease refractory to at least one cycle of salvage chemotherapy containing high-dose Ara-C; or (iv) patients with multiple relapses. Induction and re-induction chemotherapy were given according to the two institutions’ preference. All donor/recipient pairs were typed at the allelic level (HLA-A, HLA-B, HLA-Cw, HLA-DRB1, and HLA-DQB1). Transplantation was performed in case of either fully matched sibling donor (MSD) or 9–10 out of 10 matched unrelated stem cell donors (match, MUD) or haplo-identical-related donors (Haplo). Exclusion criteria were patients with active leukemic infiltration of the central nervous system, AML with M3 subtype, serum creatinine above 1.0 mg/dL, creatinine clearance less than 60 mL/min, bilirubin above 1.5 mg/dL, aminotransferases or alkaline phosphatase above 2.5 times the upper normal limit, acute or chronic heart failure, and pregnancy.

### Treatment

Patients included in the study proceeded directly to the sequential cytoreductive chemotherapy followed by allo-HSCT. The chemotherapy regimen consisted of 30 mg/m^2^/day fludarabine and 1 g/m^2^/day Ara-C for 5 consecutive days (day-21 to day-17) and idarubicin 12 mg/m^2^/day (RJH) or 10 mg/ m^2^/day (IPC) for 3 days (day-17 to day-15). No other chemotherapy was given within 3 weeks of FLAG-IDA chemotherapy, except for hydroxyurea in case of hyperleukocytosis. Seven days after chemotherapy, preparative regimen was given with 30 mg/m^2^/day fludarabine for 5 consecutive days (days −7 to −3) and 3.2 mg/kg/day i.v. busulfan for 3 consecutive days (days −5 and −3) [9]. Day 0 was designated as the day of graft infusion with G-CSF-mobilized peripheral blood stem cell for all patients. There were two different prophylaxis strategy for acute graft versus host disease (aGVHD). In RJH, the regimen included standard cyclosporine with short-course methotrexate (MTX) and mycophenolate mofetil (MMF) for MSD, while additional ATG for 4 consecutive days (days −4 to −1) was given in case of MUD (1.5 mg/kg/day) or Haplo (2.5 mg/kg/day, Table 1). As to the GVHD prophylaxis at IPC, all patients received 50 mg/kg cyclophosphamide (PT-Cy) (day +3 and +4) and cyclosporine started on day +5 alone (for MSD and 10/10 MUD) or in combination with MMF in case of 9/10 MUD or Haplo. There were also two strategies for donor lymphocyte infusion (DLI). In RJH, DLI was given in case of loss of donor chimerism, persistent, or increased minimal residual disease documented. In IPC, prophylaxis DLI (pDLI) was given for all patients without active GVHD as shown in Table 1.

**Table 1:** Patient’s characteristics

	Total	RJH	IPC	P
No. of patients	47	27	20	0.22
Sex**:** Male/female	17/30	11/16	6/14	0.18
Age: median (range)	42(16–62)	34 (16–60)	44 (26–62)	0.001
*Disease stage*				
Induction failure/early relapse	23/1	14/1	9/0	0.47
Relapse/refractory	23	12	11	
*Cytogenetic/molecular*				0.045
Favorable/intermediate/poor/not evaluable	2/18/19/8	1/9/9/8	1/9/10/0	
Previous chemotherapy median (range)	3(1–10)	4(2–10)	2(1–3)	<0.001
WBC (x10^9^/L)	3.0	4.4	3.0	0.27
Median (range)	(0.3–44)	(0.3–11.1)	(0.7–28.0)	
Circulating blast (positive/negative)	28/19	17/10	11/9	0.58
BM blast**:** median (range)	35%(1–90)	56%(1–90)	17.5%(4–79)	0.01
*Donor type*				
MSD/MUD/Haplo	13/16/16	8/11/8	5/7/8	0.76
* aGvHD prophylaxis*				
CSA + MTX + MMF ± ATG/PT-Cy + CSA ± MMF	27/20	27/0	0/20	<0.001
DLI post HSCT (yes/no)	13/34	2/25	11/9	<0.001

*RJH* Rui Jin Hospital, *IPC* Institut Paoli-Calmettes, *BM* bone marrow, *MSD* matched sibling donor, *MUD* matched unrelated donor, *Haplo* haplo-identical donor, *aGvHD* acute graft-versus-host disease, *CsA* cyclosporine A, *MTX* methotrexate, *MMF* mycophenolate mofetil, *ATG* anti-thymocyte globulin, *PT-Cy* posttransplant cyclophosphamide, *DLI* donor lymphocyte infusion

### Clinical outcomes, toxicity, and GVHD assessment

The study endpoints included engraftment, clinical response after transplantation, OS, event-free survival (EFS), relapse, non-relapsed mortality (NRM), acute GVHD, and chronic GVHD. Time to neutrophil recovery was defined as the first of 3 consecutive days in which the absolute neutrophil count exceeded 0.5 × 10^9^/L and engraftment failure as an absolute neutrophil count was above 0.5 × 10^9^/L at day +42 after allo-HSCT. Leukemia response rate was evaluated at day +30, +60, and +90 and 6, 12, 18, and 24 months after the transplant. The electronic medical record was reviewed to grade toxicities according to the National Cancer Center Common Toxicity Criteria (NCI-CTC) Version 2.0. GVHD was evaluated according to the Seattle standard criteria [10].

### Statistical methods

All data were collected from patients’ chart with last follow-up at March 31, 2017. All data was locked in April 2017. The statistical analysis was performed in the Shanghai Clinical Research Center (SCRC). OS and EFS were calculated using the Kaplan–Meier method. OS was defined as the time from allo-HSCT to death, regardless of the cause. EFS was defined as survival without event, such as no clinical response after transplantation, disease relapse, or death due to NRM. The probability of NRM and relapse were analyzed with cumulative incidence competing risk (CICR). We defined NRM as death with no evidence of leukemia relapse or progression. Univariate analysis of potential prognostic factor associated with OS, EFS, and RR and NRM were performed by log-rank test and Gray’s test. For multivariate analysis, the Cox models and the logistic regression model were built by testing the following covariates: treatment center (RJH versus IPC), donor type (MSD and MUD versus Haplo), patient age (<40 years versus ≥40 years), number of pre-transplantation chemotherapy (<3 cycles versus ≥3 cycles), disease stage (primary refractory versus relapse/refractory), and bone marrow blast (<35% versus ≥35%). The Cox models always kept the transplantation center as a variable even if its coefficients were not significant. Other covariates were selected by the backward elimination method and were held in the Cox models if the *p-*value was <0.05. SPSS (SPSS Inc., Chicago, IL) and R version 3.4.1 software packages were used for data analysis [11].

## Results

### Patient and donor characteristics

The characteristics of the patients and the donors are summarized in Table 1. The median age of recipients was 42 years (range, 16–62). AML cytogenetic status classified according to the European Leukemia Net was favorable in two patients (4.3%), intermediate in 18 (38.3%), adverse in 19 (40.4%), and 8 (17%) not evaluable. A total of 22 patients (46.9%) had induction failure, 24 (51.0%) with relapse/refractory disease, and only 1 patient (2.1%) with early relapse disease during consolidation chemotherapy. The median marrow blast percentage was 35% (1–90%), while only 3 patients had <5% leukemic blasts in the bone marrow. The median peripheral white blood count (WBC) was 3.0 × 10^9^/L (0.3–44.0), while 28 patients had circulating blast. The donors were HLA-matched sibling (MSD, *n* = 13, 27.7%), and unrelated (MUD, *n* = 18, 38.3%) and haplo-identical siblings (haplo, *n* = 16, 34.0%).

### Engraftment

All patients developed pancytopenia after the sequential conditioning regimen. Forty-three engrafted and four died in aplasia. The median time to neutrophil recovery (>0.5 × 10^9^/L) was 17 days (range, 13–56). A total of 35 patients had platelet recovery (>20 × 10^9^/L) with a median time of 21 days (range, 12–60), while eight patients failed to obtain platelet recovery.

### Disease response and outcome

At day +28, 43 patients were evaluable for response and 42 patients achieved bone marrow remission, including 32 with complete remission (CR) and 10 without platelet recovery (CRp), while only one patient failed to obtain bone marrow remission. Thus, the overall remission rate was 89% (95% confidence interval: 76–96%).

**Fig. 1:**
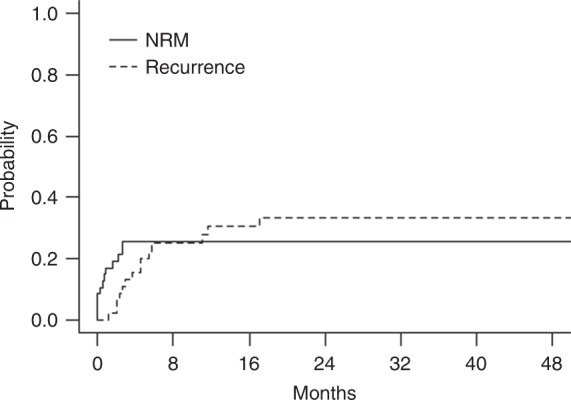
Cumulative incidence of relapse and NRM for all patients

**Fig. 2:**
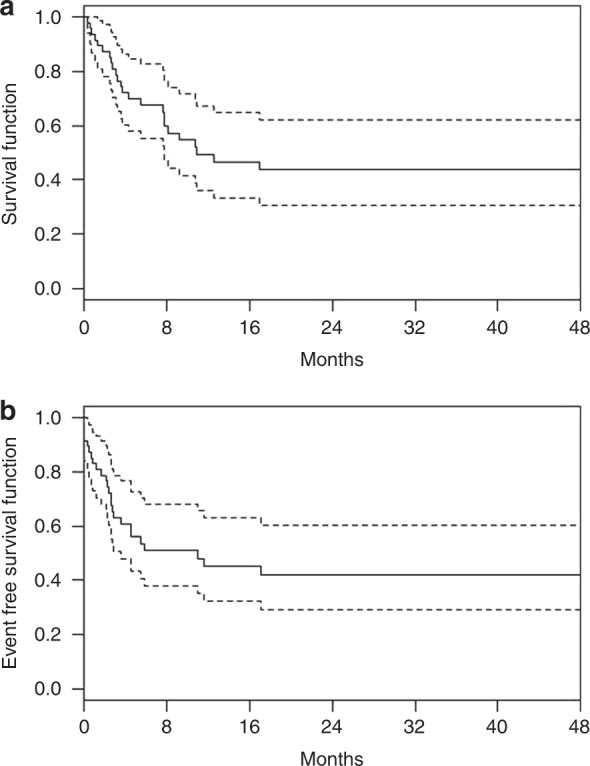
Kaplan–Meier curves with 95% confidence bound for overall survival (**a**) and event-free survival (**b**)

With a median follow-up of 24.3 months (range, 1.5–70.0), 13 patients relapsed with estimated 3-year accumulated relapse incidence at 33.5 ± 5.7% (Fig. 1). The median time to relapse was 5.1 months (2.2–18.0) after transplantation. Of note, most relapse events occurred within first 6 months and only three patients relapsed beyond 6 months (at 6.5, 12, and 18, respectively) after transplantation. A total of 12 patients died due to NRM, with 3-year estimated NRM at 25.7 ± 4.2% (Fig. 1). Overall, of the 47 patients included in the study, 24 died eventually either due to disease relapse (*n* = 12) or NRM (*n* = 12), with median survival of 10.9 months for all patients. The Kaplan–Meier estimates of OS and EFS at 3 years were 43.8 ± 7.8% and 42.3 ± 7.8% (Fig. 2).

### Effect of treatment centers on transplantation outcome

Since the patients series from the participant centers varied significantly in age, pre-transplantation chemotherapy, bone marrow blasts, GVHD prophylaxis regimen (Standard CsA-based regimen versus PT-Cy-based regimen), and pDLI post-transplantation as shown in Table 1, we compared the overall transplantation outcome between the two centers (RJH versus IPC). Of note, the OS, EFS, NRM, and RR were comparable between RJH and IPC, thus confirming the efficacy and feasibility of the transplantation protocol consisting of sequential intensive chemotherapy and reduced intensity conditioning regimen as reported previously, and also demonstrated that similar outcome can be achieved in more elderly, but less pre-treated with different GVHD prophylaxis regimens (Table 2). The effect of treatment centers on EFS was further ruled out in multivariate analysis as shown in Table 3.

**Table 2:** Comparison of transplantation outcome between the treatment centers

	RJH (*n* = 27) (%)	IPC (*n* = 20) (%)	*p*-Value
OS	38.0 ± 9.7	52.5 ± 12.7	0.22
EFS	38.0 ± 9.7	47.8 ± 13.0	0.35
NRM	34.0 ± 9.2	15.0 ± 8.0	0.12
Relapse	28.5 ± 12.2	37.5 ± 14.3	0.74

*RJH* Rui Jin Hospital, *IPC* Institut Paoli-Calmettes, *OS* overall survival, *EFS* event-free survival, *NRM* non-relapsed mortality

**Table 3:** Univariate and multivariate analysis for risk factors for EFS

Valuables	Univariate analysis	Multivariate analysis	
	*p*-Value	Hazard ratio	*p*-Value
Disease status	0.724	–	–
Treatment center	0.411	–	–
Cycles of chemotherapy	0.608	–	–
Donor type	0.122	–	–
Bone marrow blast	0.070	6.679 (1.381, 32.305)	0.018
Age	0.143	1.036 (1.003, 1.069)	0.031

### Univariate and multivariate analysis of risk factors for transplantation outcome

To evaluate the potential factor contribution to the transplantation outcome, further univariate and multivariate analyses were performed. Patients with marrow blast over median level (≥35%) and age over 40 tended to have inferior EFS (*p* = 0.070 and 0.082, respectively, in univariate analysis), while disease status (primary refractory versus refractory/refractory disease), cycles of previous chemotherapy, donor type, and treatment center were not significantly associated with EFS. In the multivariate analysis of risk factors associated with EFS, age over 40 and bone marrow blast over 35% were associated with inferior EFS, as shown in Table 3.

As to the NRM, there was no associated risk factor identified via both univariate and multivariate analysis. For disease relapse, the only associated risk factor was age over 40 in both univariate and multivariate analysis, while all other factors were not relevant statistically, as shown in Table 4.

**Table 4:** Univariate and multivariate analysis for risk factors for relapse

Valuables	Univariate analysis	Multivariate analysis	
	*p*-Value	Hazard ratio	*p*-Value
Disease status	0.887	–	–
Treatment center	0.684	–	–
Cycles of chemo	0.640	–	–
Donor type	0.255	–	–
Bone marrow blast	0.314	6.88 (0.741, 63.899)	0.090
Age	0.032	1.06 (1.013, 1.109)	0.012

### Acute and chronic graft-versus-host-disease

Among 44 patients evaluable for aGVHD, a total of 15 patients had documented II–IV aGVHD, while five had III–IV aGVHD. In 34 evaluable patients for cGVHD, 15 had cGVHD, while 10 had extensive cGVHD. Based on different median age of patients, GVHD prophylaxis, and pDLI strategy between RJH and IPC, we further evaluated the potential risk of GVHD in two cohorts and no significant difference was documented. For extensive cGVHD, we observe only one out of patients with PT-CY as GVHD prophylaxis, while six out of 17 patients with non-PT-CY strategy (*p* = 0.07).

## Discussion

Allo-HSCT remains as the only curative therapy for patients with relapsed AML [5]. Though transplantation with conventional conditioning regimen is not encouraging with OS less than 20% for patients with PIF or after relapse [7, 12–14], the sequential transplant approach combining a short course of intensive chemotherapy followed by RIC conditioning had been evaluated and reported in several studies. In the first reported FLASM-RIC study, patients having received only two previous chemotherapy courses presented a 2-year NRM incidence of 22.2% and 2-year OS and LFD around 60%, though more heavily pre-treated patients do poorly even after transplantation with 2-year OS and LFS less than 25% [8]. In a more recent report of clofarabine-based and Ara-C-based chemotherapy followed by i.v. busulfan-based conditioning regimen in AML patients with induction failure, 2-year NRM rate was 12% with no grade IV non-hematologic adverse events. A total of 75% patients achieved CR by day +30 with 2-year OS at 38%, which was superior to the 20–30% reported after most standard allo-HSCT protocol [15]. Similarly, Middeke et al. reported a 2-year OS of 43% in relapsed/refractory AML after clofarabine salvage therapy and allogeneic SCT in patients achieving a response [16].

Our study was designed to test the efficacy and feasibility of intensive cytoreductive chemotherapy sequential with reduced-intensity conditioning regimen in patients with refractory AML. The estimated 3-year OS and EFS were 43.8 ± 7.8% and 42.3 ± 7.8%, which confirmed the outcome in our previous pilot study. More importantly, we did not document significance of OS and EFS between two patients series from RJH and IPC by univariate and multivariate analyses, thus confirming the feasibility of our treatment protocol.

One may consider that a limitation of our study was the significant difference in the characteristics of patient’s population from RJH and IPC in terms of age, pre-transplantation chemotherapy, GVHD prophylaxis, and pDLI strategy. On the other hand, we were individually interested to compare the real practice of refractory AML treatment in two different countries and to test the reproducibility of the treatment outcome with FLAG cytoreductive chemotherapy, sequential with Flu–Bu conditioning, in different patient series with different transplantation systems. Though due to the number of patients enrolled in the study, which may limit the statistic power, the comparable OS and EFS and more interestingly a relatively low NRM (~15%) observed in patients treated in IPC suggested that this intense chemotherapy and conditioning protocol is feasible not only in heavily treated and younger patients (RJH), but also feasible for a more elderly although less heavily treated patients’ series (IPC). Second, though it is not a study design to directly compare the standard GVHD prophylaxis to the PT-Cy-based regimen, our data support that the overall transplantation outcome in refractory AML patients was comparable and such intense treatment can be safely carried out with PT-Cy strategy, which may tend to have fewer extensive cGVHD events [17, 18].

In the risk factors analysis, we acknowledged that transplantation for young (<40) tended to have promising EFS at 58.6 ± 11.4% after allo-HSCT, which may suggest that in young patients with documented refractory AML, direct transplantation with sequential cytoreductive chemotherapy and reduced toxicity conditioning can be considered as an treatment option even without attempts of intensified salvage chemotherapy, which may achieve CR but usually with low response rate and accumulated toxicities. In more elderly patients (≥40), we demonstrated that the underlying cause of treatment failure was mostly due to higher relapsed rate (48.1 ± 13.2% versus 20.9 ± 10.8%, *p* = 0.02) rather than NRM (27.7 ± 9.0% versus 23.8 ± 9.3%, *p* = 0.081), compared with young patients. Overall, based on the survival curve of both EFS and OS reached a plateau, we may speculate that the intense sequential treatment strategy can be considered as a curative treatment for ~40% refractory AML, which was at least comparable or even better than the approaches using FLASMA or clofarabine-based regimen, sequential with reduced intensity conditioning for refractory AML.

Relapse and transplantation-related toxicity remains as the two major causes of treatment failure. As to the relapse, even with such an intense protocol, still most relapse occurred within 6 months, with only three patients relapsed after 6 months, and no relapse events were documented after 2 years after transplantation. Of interest, we also observed that non-relapse death was documented mostly early after transplantation, with no NRM event after 9 months after transplantation. These observations may indicate that intensity of our treatment regimen was relatively toxic in 25% of patients, while such an intensity approach was not sufficient to control these 33% of patients with refractory AML. Simply escalation of the intensity of our sequential treatment strategy may not be able to overcome a group of patients with very refractory disease, but to increase the NRM.

As to the potential role of pDLI, with limited number of patients who actually received pDLI in our series and all were from IPC, it is difficult to evaluate its exact role when combined with our intensified transplantation protocol. But the anti-leukemia efficacy of the sequential conditioning regimen combining FLAG-IDA chemotherapy followed by Flu–Bu3 regimen may provide also an important treatment platform to further refine the post-transplantation strategies to improve disease control and prevent relapse in refractory AML, such as pDLI or maintenance therapy with hypomethylation agents [19, 20]. Besides we speculate that new strategies such as addition of histone deacetylase inhibitor, which may potentiate the anti-leukemic effect without increased intensity of conditioning must be engaged in future optimization, particularly for high-risk patients who relapsed early even with quite intense allo-HSCT protocol [21, 22].

Though this was a study with limited number of patients, we believed that patients enrolled in this parallel analysis were unselected, representing the typical population of refractory AML, and the sequential-conditioning regimen combining FLAG-IDA chemotherapy followed by Flu–Bu3-conditioning regimen was feasible and further confirming clinical trial with larger patients’ cohort was warranted.
